# Investigation of Recombinantly Produced Endolysins Reveals a Modular Enzyme Shared by Several Enterobacteria Phages to Exhibit Broad‐Range Lytic Activity Against Different Orders of *Gammaproteobacteria*

**DOI:** 10.1002/mbo3.70293

**Published:** 2026-04-16

**Authors:** Tatjana Kazaka, Nikita Zrelovs, Inara Akopjana, Janis Bogans, Juris Jansons, Andris Dislers, Andris Kazaks

**Affiliations:** ^1^ Latvian Biomedical Research and Study Centre Riga Latvia

## Abstract

Endolysins or murein hydrolases are hydrolytic enzymes produced by bacteriophages to cleave the host's cell wall during the final stage of the lytic cycle. Whereas globular endolysins are composed of a single enzymatically active domain (EAD), modular endolysins have at least two recognizable modules, often comprising a cell wall binding domain coupled to an EAD. Although such enzymes seem to be rarer, their activity often exceeds that of their globular counterparts. Here, we explored five previously uncharacterized modular endolysins for their expression, purification, and activity against a panel of distinct environmental Gram‐negative bacteria. Out of the selected endolysins derived from Enterobacteria‐infecting phages, two were soluble and were purified to near homogeneity. Among them, endolysin shared by several Enterobacteria phages, referred to as El1, exhibited a notable bacteriolytic activity not only from within, but also from without the cells. The effects of the studied enzyme on bacterial growth and viability were studied in detail in *Escherichia coli*. Visual inspection of the treated cells verified that the enzyme could penetrate the *E. coli* cell membrane when applied exogenously. Interestingly, El1 was active in the absence of ethylenediaminetetraacetic acid (EDTA) against multiple environmental Gram‐negative bacteria representing different *Gammaproteobacteria* orders. Although the exact Gram‐negative lysis mechanism of El1 remains unknown, the breadth of its target range suggests El1 as a promising candidate for future studies to scrutinize natural endolysin interactions differences against evolutionary distinct bacteria.

## Graphical abstract

The recombinant endolysin El1 derived from Enterobacteria bacteriophages showed potent bactericidal activity against Gram‐negative bacteria when applied exogenously, even in the absence of membrane‐permeabilizing agents, such as ethylenediaminetetraacetic acid (EDTA).

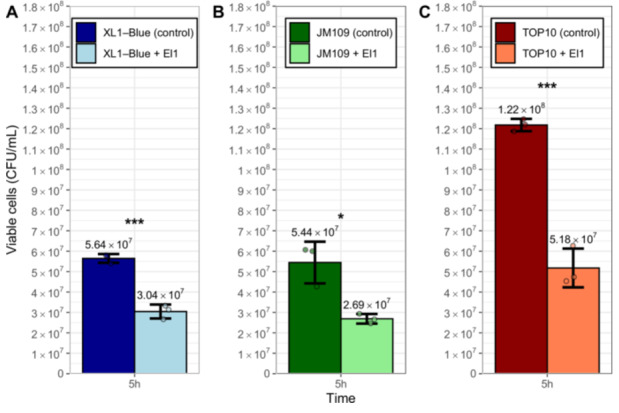

## 1 Introduction

Endolysins or murein hydrolases are hydrolytic enzymes produced by bacteriophages to cleave the host's cell wall during the final stage of the lytic cycle to release the phage progeny into the surrounding environment, resulting in the death of the infected cell. In spite of the conserved biological function of endolysins, these enzymes are very diverse in their structure and specificity. Generally, two structural classes of endolysins are recognized, designating each endolysin as either a globular or a modular endolysin. Whereas globular endolysins are composed of a single enzymatically active domain (EAD), modular enzymes have at least two modules, mostly also comprising a cell wall binding domain (CBD) besides an EAD (Briers and Lavigne 2015).

Endolysins were first used therapeutically in 2001 (Nelson et al. 2001) and are now being considered as promising antibacterial agents due to their high effectiveness and specificity in comparison with antibiotics, usability of which is becoming limited due to the rise in antimicrobial resistance prevalence (Fischetti 2008). Exogenous application of endolysins has mainly been found effective against Gram‐positive bacteria due to ease of endolysin access to their substrate (Nelson et al. 2001; Schuch et al. 2002; Schmelcher et al. 2012). Gram‐negative bacteria, on the other hand, possess an outer membrane that prevents extracellular lysin molecules from reaching and digesting the peptidoglycan (PG) layer of the cell.

Only a fraction of naturally occurring endolysins has been demonstrated to be effective against Gram‐negative pathogens, such as *Acinetobacter baumannii* (Lai et al. 2011; Lood et al. 2015) and *Pseudomonas aeruginosa* (Walmagh et al. 2012). The ability of such endolysins to naturally permeate the bacterial outer membrane is hypothetically attributable to the mechanisms that likely involve amphipathic or positively charged protein regions, but are not understood in mechanistic details. Thus far, several distinct approaches for outer membrane destabilization and endolysin delivery to the PG layer have been proposed, including formulations containing membrane‐permeabilizing agents (Briers et al. 2011) or antibiotics (Thummeepak et al. 2016), fusion of the endolysins to the phage receptor‐binding proteins (Zampara et al. 2020), as well as endolysin encapsulation within liposomes (Bai et al. 2019). Endolysin fusions with outer membrane‐permeabilizing antimicrobial peptides (“artilysation”) provide another highly promising approach for endolysin‐based drug design, demonstrated by the efficacy of the recently developed Artilysin Art‐175 (Lysando, Germany) active against *A. baumannii*, *P. aeruginosa*, and colistin‐resistant *Escherichia coli* (Schirmeier et al. 2018).

Very recently, we have demonstrated that “artilysation” of a globular endolysin derived from the phage Enc34 (Kazaks et al. 2012; Cernooka et al. 2022; Cernooka et al. 2024) with antimicrobial peptides leads to its improved activity against Gram‐negative bacteria (Kazaka et al. 2024). In the frame of the same project, we have additionally explored several other naturally occurring yet previously uncharacterized modular endolysins in terms of their effects against Gram‐negative bacteria. Although modular endolysins are substantially rarer compared with their globular counterparts, their activity was demonstrated to exceed the activity of globular enzymes up to an average of fivefold (Briers et al. 2009; Walmagh et al. 2013). Thus, characterization of different natural modular endolysins and evaluation of their activity against Gram‐negative bacteria is a promising area of research that is expected to improve the knowledge base for naturally occurring endolysin improvement and artificial endolysin design.

In this study, we focus on the recombinant production and activity characterization of five distinct, previously unstudied, modular endolysins naturally found in bacteriophages infecting Enterobacteria. One of the studied proteins, an endolysin shared by several Enterobacteria phages, designated here as El1, proved to be amenable to recombinant production and demonstrated efficacy against multiple distinct environmental Gram‐negative bacteria, warranting further in‐depth studies on its mechanism of action and potential applicability against bacterial pathogens of healthcare or economic importance.

For this study, we selected five natural modular endolysins from previously published bacteriophage genomes, and aimed to explore them in terms of recombinant expression, protein solubility, and lytic potential against a broad panel of Gram‐negative bacteria. Previous experimentation in the lab gave a hint that particular endolysins could efficiently lyse *E. coli* cells from within when produced recombinantly (in the absence of other usual Gram‐negative‐hosts‐infecting phage lysis module proteins, such as holins or spanins), such endolysins have partially accumulated in the expression medium (Kazaka et al. 2024). In the end, a single endolysin—El1 was selected for a detailed investigation. When El1 was applied exogenously to a range of Gram‐negative bacteria (laboratory *E. coli* strains, a diverse set of environmental *Pseudomonas* spp., as well as available strains from other genera, spanning at least four different orders of *Gammaproteobacteria*), inhibition of cell growth in a spot test with varying endolysin concentrations (0.35, 0.7, 1.4, and 2.8 mg/mL) was registered for many of the strains. The effects of El1 on cell growth and reduction of viable cell number were investigated in finer detail against common laboratory *E. coli* strains, revealing highly varying effects on different strains of the same species. The current study is severely limited by the lack of a detailed understanding of the molecular mechanisms of El1 action against a range of Gram‐negatives. Nevertheless, due to very easy purification, high stability, and broad antibacterial action, we suggest that both the studies and informed improvement of El1 or similar endolysins could be pursued to evaluate their potential to be added to the arsenal of antibiotic alternatives that might help in the ongoing battle against drug‐resistant Gram‐negative bacteria.

## 2 Materials and Methods

### 2.1 Plasmid Constructions

Codon‐optimized genes for the expression of five modular endolysins (UniProt entries W8JPH7, G0XNW2, A0MZE3, A0A0U2I1S0, and A0A142IIL8) in *E. coli* were designed by GenScript and generated by gene synthesis at BioCat GmbH (Heidelberg, Germany). The respective genes for the recombinant production of the desired endolysins (designed as El1 to El5), respectively, were subsequently cloned into the pET24a(+) vector (Novagen) using *Nde*I and *Xho*I restriction sites. All these constructs were designed for the resulting protein to contain the MGSH_6_GS sequence at the N‐terminus. This artificial sequence contains six histidines for Ni‐affinity purification separated by short GS linkers at both sites. The second GS‐encoding sequence also contains the *Bam*HI restriction site. Circular vector maps, as well as the full codon‐optimized sequences of studied proteins, alongside their translation and locations of the associated conserved domains (as identified by the web‐based National Center for Biotechnology Information [NCBI] CD‐search under default settings), are shown in Figures S1 and S2.

### 2.2 Expression Conditions

Expression plasmids were transformed into *E. coli* strain BL21 (DE3) cells. For inoculum, the cells were cultivated without shaking overnight at +37°C in a selective Luria–Bertani (LB) medium (10 g/L tryptone, 5 g/L yeast extract, 10 g/L NaCl, pH 7.2) containing 30 µg/mL kanamycin. To start protein synthesis by lactose auto induction, the cells were transferred to 400 mL of selective 2xTY medium (16 g/L tryptone, 10 g/L yeast extract, 5 g/L NaCl, pH 7.2) with the following additives: 60 mL of phosphate solution (23.13 g KH_2_PO_4_, 125.42 g K_2_HPO_4_, dH_2_O until 1 L, pH 7.4), 0.75 mL of 40% glucose, 1 mL of 80% glycerol, 5 mL of 10% lactose, and 0.4 mL of 1 M MgSO_4_. Expression was performed in Erlenmeyer flasks on a shaker with 200 rpm at +37°C overnight (16–20 h). Both the concentration of lactose and the induction time were established in our previous experiments. Longer incubation times were noted to lead to a decrease in the target protein recovery. After low‐speed centrifugation (1500*g* for 10 min), cell and medium fractions were collected and analyzed for the presence of the target protein (Kazaka et al. 2024).

### 2.3 Protein Purification

The first step of protein purification was done according to the protocol previously published in Kazaka et al. (2024). For intracellular protein purification, 1 g of wet cells was disrupted in 6 mL of lysis buffer (20 mM Tris–HCl, 300 mM NaCl, pH 8.0) by sonication, and soluble proteins were separated after centrifugation for 30 min at 18,500 *g*. The proteins were purified using Ni‐ion affinity chromatography on the HiTrap IMAC FF column (Cytiva). The supernatant was applied (1 mL/min) on a column containing 1 mL beads equilibrated with a lysis buffer. After washing the column with 10–20 mL of lysis buffer with 10 mM imidazole, the protein was eluted with elution buffer (20 mM Tris–HCl, 0.5 M NaCl, 0.5 M imidazole, pH 8.0) in 1 mL fractions using a linear gradient (10 CV).

Alternatively, for purification of the extracellular protein, 400 mL of expression medium without cells was passed through a HisTrap Excel 5 mL column (Cytiva) in a lysis buffer at 5 mL/min and washed with 50 mL of 10 mM imidazole buffer. The target protein was collected as a single fraction with an elution buffer. Purification was performed using the Akta Prime Plus system (Cytiva).

As a second step of purification, after Ni‐affinity column proteins were loaded onto a Superdex 200 size‐exclusion column equilibrated with phosphate buffer in 0.5 M saline and separated at 2 mL/min. Purified proteins representing major peaks were then adjusted to 2 mg/mL stock solutions using an Amicon 10 kDa molecular weight cut‐off filter device (Millipore) and stored at +4°C until use. Concentration of the purified proteins was determined by measuring the absorbance at 280 nm (*A*₂₈₀) and dividing it by the molar extinction coefficient (*ε*), according to the following formula: *C* = *A*₂₈₀/*ε*.

### 2.4 Activity Assay

To evaluate the effects of our recombinantly produced endolysin exogenous application on Gram‐negative bacteria growth dynamics, *E. coli* K12 derivative strains XL1‐Blue, JM109, and TOP10 were initially used as common model organisms. For a first activity check, endolysin solution at 20 µg/mL was added to 20 mL of freshly grown cells diluted to an optical density (OD) ~0.1 (*A*_540_) in LB medium, cultivated in small flasks with orbital shaking at +37°C. The OD was measured hourly for a period of up to 5 h post‐endolysin challenge and compared with the control growth curve. Each experimental condition was also evaluated in the presence of 0.5 mM ethylenediaminetetraacetic acid (EDTA), known to destabilize the outer membrane of Gram‐negative bacteria. The experiment was performed in three independent replicates.

For a more precise test, cell viability was evaluated by the proxy of colony‐forming unit (CFU) concentration assays. For this, bacterial cells were similarly cultivated with shaking in LB medium at +37°C until an OD of 0.1 (*A*_540_). The cell suspension was next aliquoted into six tubes by 5 mL, and endolysin solution was added to three tubes until the final concentration reached 20 µg/mL. In the three control tubes, the same volume of phosphate‐buffered saline (PBS) was added. Prior to the start of the experiment and after defined time points (1, 2, 3, 4, and 5 h after mixing the cells with a product), the aliquots of the cell suspension were taken and immediately used for the preparation of the serial dilutions up to 10^5^ with a step of 10^1^ using PBS, 50 µL of which were spread on LB agar plates afterwards. The viable cell titer (CFU/mL) in the samples at the given time points after the addition of the product was derived from the number of CFUs observed after overnight incubation at +37°C, the dilution factor, and the volume of the dilution used. Cell viability assays were performed in independent triplicate.

The viable cell count titer reduction (% of CFU/mL) alleged to the bactericidal activity of a product was evaluated at each different time point post‐addition as follows: average viable cell titer in the respective treatment group was divided by an average cell titer in the control group and the percentage of change was obtained by subtracting this fraction from 100% (representing control group).

As a third method to detect the presence or absence of studied endolysin activity in a higher throughput, we used spot testing against a range of locally isolated Gram‐negative environmental bacteria readily available in the lab in connection with the other projects we are involved in. The bacterial strains used in the assays were previously identified by querying their near‐complete 16S ribosomal RNA (rRNA) gene sequence (obtained from Sanger‐based sequencing using universal primers 27F and 1492R) against the aggregated 16S rRNA sequence EzBioCloud database and recording the closest match. The assays involved pipetting of serial dilutions of the endolysin (resulting in final concentrations of 0.35, 0.7, 1.4, and 2.8 mg/mL) onto a double‐layer agar Petri plate seeded with a desired bacterial strain lawn in the top agar layer. For each concentration tested, 10 µL was spotted. In the case of lysis, a phage‐like lawn clearance zone was observed upon visual inspection at the place of the spot after an overnight incubation at +37°C. The presence or absence of the lysis zones was recorded in a binary form (lysis/no lysis) for each targeted strain and endolysin concentration tested.

### 2.5 Electron Microscopy

Bacterial cells were absorbed from the suspension onto carbonized Formvar‐coated 300 Mesh copper grids (Agar Scientific) through a 5‐min incubation. Subsequently, the grids underwent rinsing with a 1mM EDTA solution at pH 8.0, staining with a 0.5% uranyl acetate solution for 1 min, and air drying. The stained grids were analyzed using a JEM‐1230 electron microscope (JEOL) operating at an accelerating voltage of 100 kV, and images were captured with the MORADA digital camera using iTEM imaging software (Olympus).

### 2.6 El1 Relation to Other Endolysins

The nonredundant NCBI Protein database was queried using the El1 sequence (Accession No. AXF39283.1), restricting the search to the sequences originating from *Caudoviricetes* phages (taxid 2731619) using protein–protein basic local alignment search tool (BLASTP) search via an online suite (Altschul et al. 1990). The top 100 highest‐scoring hits were downloaded and locally clustered at 95% global identity using cd‐hit v4.8.1 (Li and Godzik 2006). The El1 sequence was added to the resulting representative evolutionary context sequences, and multiple sequence alignment (MSA) was performed using MAFFT v7.453 using the accurate mode (Katoh and Standley 2013). Next, the resulting MSA was used as an input for the evolutionary relationship reconstruction using IQ‐TREE v2.0.6 (Nguyen et al. 2015) via maximum‐likelihood phylogenetic analysis (using automatic evolutionary model selection via ModelFinder (Kalyaanamoorthy et al. 2017) and assessing branch supports using 1000 ultrafast bootstrap (UFBoot) replicates (Minh et al. 2013). The resulting tree was visualized using FigTree v1.4.4 (Rambaut 2018).

### 2.7 Statistical Analysis

The statistical significance of the effects of El1, El5, and EDTA, alone or in combination, on *E. coli* strains XL1, TOP10, and JM109 was assessed for the growth curve, assuming *α* = 0.05. For each strain in the growth curve experiment, the area under the curve was calculated for each of the three independent replicates per strain over 5 h using the trapezoidal rule (trapz, pracma R package; https://cran.r-project.org/web/packages/pracma/index.html) and normalized to that of the untreated control. One‐way analysis of variance (ANOVA) was performed, with homogeneity of variances and normality of residuals checked using Levene's and Shapiro–Wilk tests, respectively. Pairwise differences between the experimental groups were evaluated using Tukey's honestly significant difference post hoc test, and significance groups were visualized with compact letter displays (multcompView R package, https://cran.r-project.org/web/packages/multcompView/index.html).

Viable cell count reduction experiment data were also analyzed to determine whether there are significant differences between the untreated controls and El1 treatment groups (without the addition of EDTA) in the case of the same *E. coli* strains XL1, TOP10, and JM109, assuming *α* = 0.05. For each strain in the CFU reduction experiment, raw colony counts were adjusted for dilution factors, and transformed to reflect absolute cell concentrations. Statistical analyses were performed separately at each time point (1–5 h) for each strain. One‐way ANOVA was used to assess whether the differences in CFU among treatments are statistically significant, with Levene's test applied to verify homogeneity of variances and the Shapiro–Wilk test to assess normality of residuals.

In both cases, results of the statistical analyses were integrated into the corresponding visualizations of the results using the ggplot2 package (https://ggplot2.tidyverse.org). Statistical analyses were conducted in RStudio (v1.3.959, http://www.posit.co/).

## 3 Results and Discussion

### 3.1 Expression, Purification, and Characterization of Five Modular *E. coli* Phage Endolysins

The vast majority of endolysins from phages targeting Gram‐negative bacteria are globular, consisting of a single EAD. The remaining few endolysins, however, are composed of an EAD and an additional CBD, and are, thus, defined as modular. In this work, we have decided to focus on modular endolysins targeting *E. coli*, since the presence of CBD has been demonstrated to enhance their lytic activity (Briers and Lavigne 2015). A previously conducted thorough database search has revealed five potential objects, one of which contains the PG‐binding domain 1 (UniProt ID W8JPH7; El1) and the other four contain the PG‐binding domain 3 (UniProt IDs: G0XNW2, A0MZE3, A0A0U2I1S0, A0A142IIL8; El2‐4, respectively) alongside either a *N*‐acetylmuramidase or a glycosylhydrolase conserved EADs, respectively (Figure S2 and Table S1). These sequences were retrieved from NCBI's Protein database using a plain text query‐based strategy that searches all fields of the database entries for “*Escherichia coli*” and either “PG_binding_1” or “PG_binding_3,” with “viruses” as a filter.

**Figure 1:**
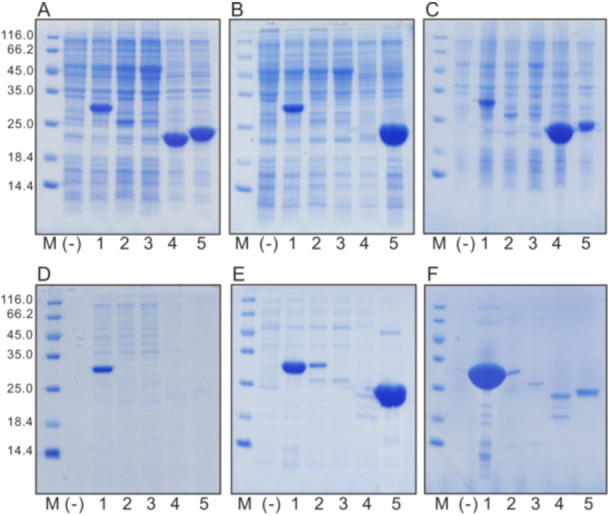
Coomassie‐stained SDS polyacrylamide gel with 5% stacking and 15% separating gel demonstrating synthesis and localization of endolysins El1–El5 expressed in *Escherichia coli* cells. (A) Total cell proteins, (B) soluble cell fraction, (C) cell debris fraction, (D) protein content in expression medium, (E) Ni‐affinity purified target intracellular protein, and (F) Ni‐affinity purified target protein from expression medium. M, molecular mass marker; (‐), negative control. Track numbers 1–5 correspond to El1–El5, respectively. SDS, sodium dodecyl sulfate.

We have established a production system for all five of these proteins, according to standard protocols previously successfully developed in our lab. Hexahistidine tags were added at the N‐terminus to facilitate protein purification. First, the proteins were screened for their synthesis levels and solubility. High‐level synthesis was evident by Coomassie‐stained polyacrylamide gel (PAAG) for endolysins El1 (Mw 29.9 kDa), El4 (21.4 kDa), and El5 (23.7 kDa); however, traces of expression were also detectable for El2 (23.8 kDa) and El3 (23.8 kDa) (Figure 1A). Out of them, only El1 and El5 were found in the soluble fraction after cell lysis by sonication (Figure 1B).

El2–El4 were found completely insoluble as they appeared in the cellular debris fraction (Figure 1C). For El2 and El3, this might be due to the presence of transmembrane regions located in the N‐terminus of the molecule. For El4, no transmembrane regions were detected, so insolubility should be explained by other reasons, for example, aggregation. We also checked the expression medium for the presence of target proteins and, surprisingly, found there was a large amount of El1 (Figure 1D), which indicates strong cell lysis. Normally, in the case of phage‐driven cell lysis from within, the synergic action of at least two phage proteins—holin and endolysin—is necessary (Young 2013). However, for some of the cloned endolysins, holin‐independent cell lysis has been observed in this and other works. Interestingly, in one particular case, the export of endolysin was caused by an N‐terminal transmembrane domain (Xu et al. 2004). However, El1 described in this work is free from identifiable transmembrane sequences; thus, cell lysis is expected to be mediated by other mechanisms that remain yet to be elucidated in further work.

A purification experiment by Ni‐affinity chromatography was performed to remove carryover of undesired contaminants. As expected, only El1 and El5 could be purified from the intracellular protein pool (Figure 1E), whereas in the case of the expression medium purification, only El1 appeared to bind to Ni‐beads (Figure 1F). This allowed us to reason in favor of shifting our focus further to two proteins only. Thus, El1 and El5 were selected as candidates for further experiments evaluating their activity upon exogenous application to Gram‐negative bacteria.

**Figure 2:**
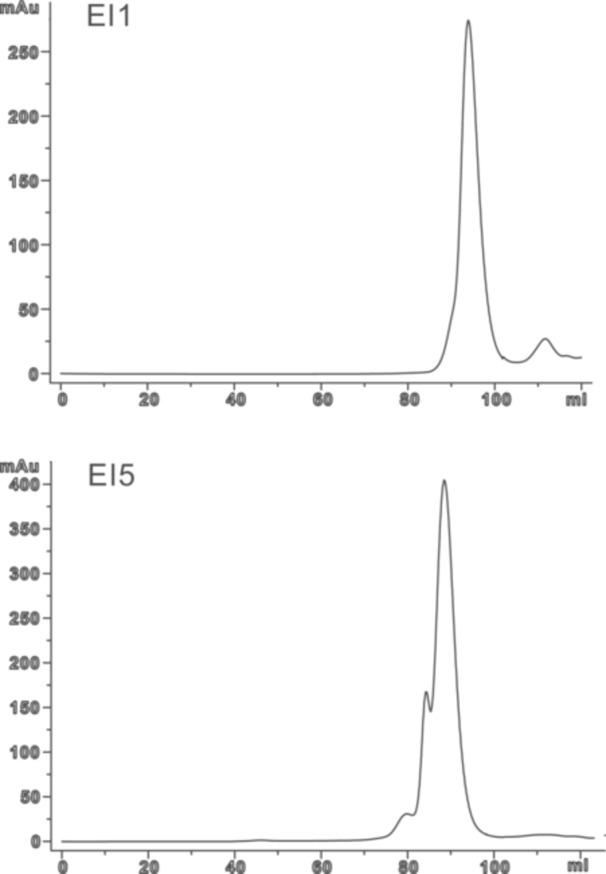
Size‐exclusion chromatography profiles for El1 and El5 endolysins. Although El1 has a higher Mw, El5 elutes faster, indicating possible dimerization.

Both selected target proteins were loaded onto the size‐exclusion chromatography column for final polishing, as well as for the aggregation test. The elution profiles revealed that the El1 protein appeared in a monomer fraction (Figure 2, top). In contrast, the elution profile of El5 suggested its possible dimerization (Figure 2, bottom). These observations were in line with the 3D structures of their close homologs, a monomeric endolysin from *Burkholderia* phage AP3 (Maciejewska et al. 2017) resembling El1 and a dimeric typhoid toxin secretion‐associated muramidase from *Salmonella* (Geiger et al. 2020) showing similarity to El5, respectively. Although small amounts of more complex structures, such as trimers and tetramers, were noted for El5, no accented aggregate peaks could be observed in both El1 and El5 cases. Taken together, the data suggested the absence of free cysteines on the surface of El1 and El5 molecules and indicates the correct folding of both proteins.

As for the production amounts, concentrations of 40–50 mg/L are readily achievable for both proteins described in this work. The important difference, however, lies in the fact that El1 is better purified from the expression medium, whereas El5—as an intracellular protein. According to the Coomassie‐stained PAAG, both proteins reach a relative purity of 90%–95% after gel filtration.

### 3.2 Bacteriolytic Properties of El1 and El5 Proteins

**Figure 3:**
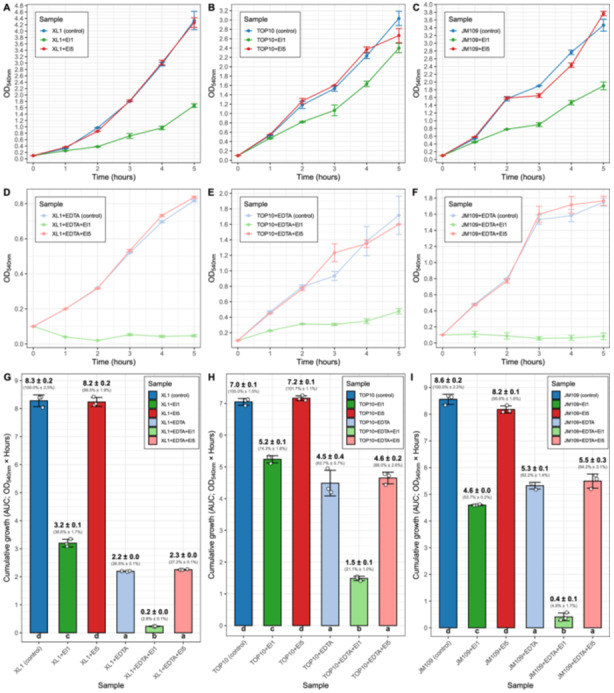
Effects of the studied endolysins El1 and El5 on the growth of the laboratory *Escherichia coli* strains in the presence or absence of ethylenediaminetetraacetic acid (EDTA). Tiles (A)–(C) show the effects of the El1 and El5 on *E. coli* XL1‐Blue, TOP10, and JM109 without EDTA, respectively. Tiles (D)–(F) show the effects of the El1 and El5 on the *E. coli* XL1‐Blue, TOP10, and JM109 in the presence of EDTA, respectively. Individual data points in tiles (A)–(F) represent the mean OD_540nm_ value of three independent replicates, and the error bar represents 1 SD. Tiles (G)–(I) show the differences in cumulative growth (represented by the area under the curve [AUC], OD_540_ × hours) between experimental groups for *E. coli* XL1‐Blue, TOP10, and JM109. Bar heights in tiles (G)–(I) show the mean AUC of three individual replicates per experimental group; the error bars indicate 1 SD. Absolute AUC values, as well as AUC percentages normalized against the control group, are indicated above the bars ±1 SD. Letters below the bars represent significance groups as determined by Tukey's honest significance test (*α* = 0.05).

To compare the bacteriolytic activity of purified El1 and El5 on bacterial cells from without, first, *E. coli* K12 derivative strains XL1‐Blue, JM109, and TOP10 were incubated with equal amounts of endolysins (20 µg/mL), and changes in OD were monitored hourly for 5 h post‐endolysin addition (Figure 3).

While El5 had practically no impact on the growth of selected *E. coli*, El1 clearly exhibited a notable effect on cell growth in all three of the strains tested. Interestingly, there was a variability in the magnitude of the observed El1 effect across the strains. The most pronounced effect was observed in the case of XL1‐Blue cells (Figure 3A), cumulative growth of which has been reduced by an average of more than 60% (Figure 3G). In the case of JM109 and TOP10, cell growth was also significantly affected by the presence of El1 (Figure 3B,C), but resulted in a cumulative growth reduction to a smaller extent than that of XL1, when normalized against the control (Figure 3G–I).

It is well known that the addition of EDTA is highly synergistic with the presence of defined endolysins or their derivatives (Sisson et al. 2024), in addition to having a detrimental effect on the growth of bacterial cells on its own. Therefore, another round of experiments was performed in the presence of 0.5 mM EDTA (Figure 3D–F). Notably, EDTA itself has strongly influenced the cell growth, resulting in an average of 5‐h cumulative growth representing 26.5%, 63.7%, and 62.2% of the untreated control for XL1, TOP10, and JM109, respectively (Figure 3G–I). However, the presence of EDTA has notably amplified the effects El1 exerted on all three strains, leading to near‐complete inhibition of cell growth in the case of XL1 and JM109 (Figure 3D–I). For instance, there is less than a threefold reduction in the cumulative growth between the control and application of endolysin alone in the case of XL1 cells. Whereas comparison of EDTA‐treated cells versus EDTA + El1 group reveals a 10‐fold difference in cumulative growth between the two (Figure 3G). The same synergistic effect of El1 and EDTA applies to TOP10 and JM109 cells, as well, again revealing amplification to different degrees, showing how variable the effects of the El1 treatment can be even in the case of highly closely related strains representing the same species.

Interestingly, El5 had no significant effect on cumulative cell growth, neither when applied alone (no differences with the untreated control) nor when applied together with EDTA (no differences with the group treated with EDTA alone). We have no rational explanation why one endolysin works better than another; apparently, this is a combination of structural and functional properties that make each endolysin unique. El1 and El5 have different CBD domains (PG‐binding 1 and PG‐binding 3, respectively), which might affect the bacteriolytic effect. However, we have also tested some El1 analogs, and none showed a comparable effect to El1 (our unpublished observations). Definitely, this fact needs extensive additional investigation.

**Figure 4:**
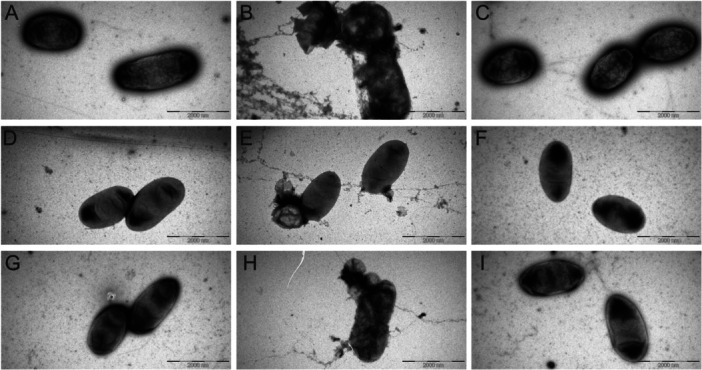
Transmission electron micrographs demonstrating the effects of the endolysins El1 and El5 on the integrity of *Escherichia coli* cells. (A–C) XL1‐Blue cells, (D–F) TOP10 cells, and (G–I) JM109 cells. A, D, G, intact cells; B, E, H, cells treated with El1; C, F, I, cells treated with El5.

To see the aftermath of the endolysin application on the integrity of the cells, cell samples treated with an endolysin without the presence of EDTA were collected 5 h after the addition of endolysin, and were analyzed by electron microscopy (Figure 4). While not attempting quantification of the effects for practical reasons, during the inspection of the samples, it became obvious that El1 has had a dramatic effect on the integrity of the randomly encountered cells in different fields of view on the grids. At the same time, in the case of El5 treatment, practically no effect on cell morphology could be noted when compared with the control samples. However, it is worth noting that the electron microscopy investigations performed here were not fit for faithful quantification and merely represented inspection of a series of images taken in different fields of view (five for each sample) from which three of the most representative ones were selected by an operator to create a collage demonstrating differences between the experimental groups. Additionally, it should be noted that only a fraction of cells were affected in the presence of El1, while others remained intact (which was to be expected based on the growth curve assays).

**Figure 5:**
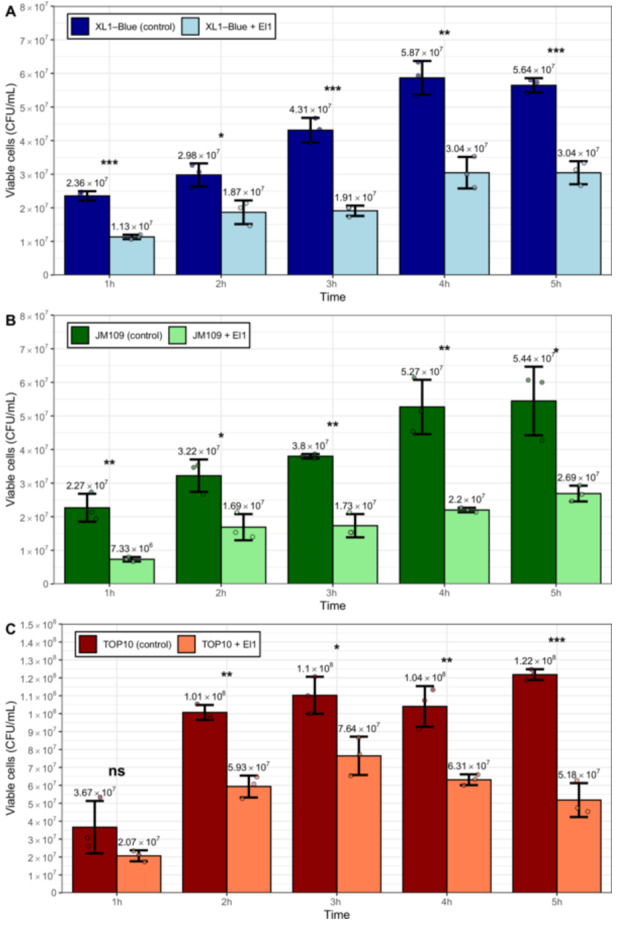
Impact of the El1 endolysin on the viable cell titers of different Escherichia coli strain cells. Effects of the El1 addition on the E. coli strains: top tile (A), XL1‐Blue; middle tile (B), JM109; bottom tile (C), TOP10. In all tiles, the numbers above the bars indicate the average CFU/mL for the respective group from three independent replicates. Bar heights correspond to the mean of the three replicates, and error bars indicate ±1 SD. Jittered points represent the average values of the three technical replicates for each independent experiment, respectively. Symbols above the bars indicate statistical significance levels based on the results of the analysis of variance (ANOVA performed for pairs of control and treatment at 1, 2, 3, 4, and 5 h separately at α = 0.05) as follows: nsp ≥ 0.05; *p < 0.05; **p < 0.005; ***p < 0.0005. CFU, colony‐forming unit.

To investigate the actual number of fatally damaged cells and quantify the endolysin effects on cell viability in a more straightforward way, we performed a CFU assay similar to that described in our previous manuscript (Kazaka et al. 2024), but with some modifications outlined in Section 2. The difference in the numbers of viable cells due to the bactericidal effect of the exogenously added endolysin application was statistically significant already after 1 h of incubation for two out of three reference strains (except in the case of TOP10 cells). The differences in the CFU counts between the El1 treatment group and the untreated control were statistically significant for all three strains in samples quantified at 2–5 h (Figure 5). In all three strain cases, the effects of El1 on the viable cell number after 5 h resulted in survival of around half (~42.5%, ~49.4%, and ~53.9%, also dependent on the targeted strain) of the total viable cell population when compared with the untreated control. This indicates that the impacts on cumulative growth, as measured by the areas under the OD multiplied by time curves for the strains depicted in Figure 3, do not directly reflect the actual damage to the viability of the cells. A plausible explanation for this being the fact that the damaged cells, which are already unable to form colonies and have lost their viability, still remain in solution and impact the OD due to the nature of the damage to them. While we expect that this would also mean that even a greater effect would be detectable for El1 in the presence of EDTA in cell viability assays, this was not a task of the current study, as the presence of the EDTA is incompatible with many of the potential settings where endolysins could be used for bacterial biocontrol.

### 3.3 El1 Distribution in Nature

Amino acid sequence matching with El1 was reported first in 2014 by Zhang and co‐workers for *Salmonell*a myophage vB_SalM_SJ2 (UniProt entry W8JPH7; GenBank Accession NC_023856) isolated from wastewater in Indiana (Zhang et al. 2015). Next, the same sequence was found in 2018 for Shiga toxin‐producing *E. coli* phage vB_EcoM_Sa157lw (UniProt entry A0A3G1RPM5; GenBank Accession MH427377) isolated from service water in California (Liu et al. 2019). Finally, the same protein was detected in 2021 for *Escherichia* phage Sa157lw (UniProt entry A0AAE8Y2H2; GenBank Accession OK322699) isolated from surface water in a produce‐growing area (Liao et al. 2022). Although the complete genomes of the corresponding phages were submitted to GenBank, and each of them was characterized to an extent, no further experiments were apparently performed with their corresponding endolysin, to the best of our knowledge.

Given the broad host range of El1 and its relatively uncommon feature of being active against a diverse range of Gram‐negatives when applied from without, even in a standalone fashion, we decided to further look at the distribution of El1‐like sequences in nature. Besides broad representation within *E. coli* and *Salmonella* phage proteomes, we were able to find relatively close homologs of El1 (100% query coverage and identity of > 80%) in at least several phages infecting representatives of the *Shigella*, *Enterobacter*, *Klebsiella*, and *Dickeya* genera. More distantly related proteins (> 98% query coverage with 43%–65% identity) were also found to be encoded by genomes of phages infecting *Erwinia, Serratia, Pseudomonas, Ralstonia, Burkholderia*, and even *Aeromonas* species.

Notably, each of the homologs from these phages had a Muramidase (PSSMID 432137) conserved domain coupled with a discernible N‐terminal peptidoglycan recognition protein domain (PSSMID 442635) as determined by the NCBI's web conserved domain search tool (Marchler‐Bauer et al. 2011).

The isolation sources of phages harboring similar endolysins associated with the respective phage complete genome accessions were most frequently indicated as types of aquatic (wastewater, sewage, river waters), terrestrial (different types of soils), and higher organism‐associated sources (human metagenome, “kidney stone,” “lung of bronchiectasis patients,” “chicken feces,” and “dead brook trout,” to name a few).

Such a distribution of similar proteins across different Gram‐negative bacterial genera phages isolated from very different ecological contexts suggests it to be a versatile solution for the lysis of PG and might explain a broad‐range enzymatic activity of El1, hinting at its potential to be exogenously applied against a wide range of targets.

**Figure 6:**
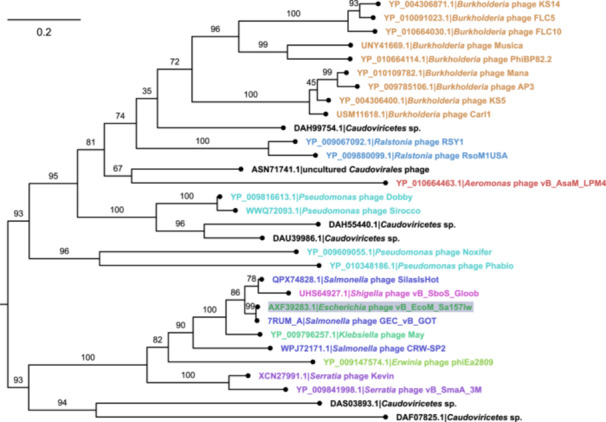
Evolutionary relationships between the El1 and representative related homologs (top 100 BLASTP hits among *Caudoviricetes*, deduplicated at 95% identity using cd‐hit). Midpoint‐rooted maximum‐likelihood tree is shown. The tree is drawn to the respective scale, and branch lengths correspond to the number of amino acid substitutions per site. Ultrafast bootstrap (UFBoot) support percentage (out of 1000 replicates) is indicated above the corresponding branches. In the tree, tips are labeled as “Respective protein accession|Originating phage,” and tip labels are colored based on the host genus of the phage encoding the respective protein; labels of the leaves containing the amino acid sequences from uncultured bacteriophages are black. Tip representing the amino acid sequence of El1 (represented by AXF39283.1 from *Escherichia* phage vB_EcoM_Sa157lw, which shares the exact same sequence as AHK61394.1 from *Salmonella* phage vB‐SalM‐SJ2, as well as UCR91132.1 from *Escherichia* phage Sa157lw, which was added to the representative clustered homolog data set) is additionally highlighted by a blue rectangle. Multiple sequence alignment used to generate the tree had 31 sequences with 349 columns, 307 distinct patterns, 204 parsimony‐informative, 58 singleton sites, and 87 constant sites. On the basis of the ModelFinder inference, WAG + F + I + G4 was used as the best‐fit evolutionary model.

A maximum‐likelihood tree placing El1 within the context of related representative endolysins is depicted in Figure 6 (clustering results of the nonredundant top 100 *Caudoviricetes* hits can be found in File S1, alongside the MSA used to generate the tree (File S2), as well as the tree itself in the newick format, including branch UFBoot supports (File S3).

### 3.4 Structural Similarity of El1 to Endolysins From Other Bacteriophages

When querying the El1 sequence against the protein data bank (PDB) using the HHpred web‐server (Söding et al. 2005) in early spring 2026, two hits with the 100% probability were noted to experimentally determined structures from proteins of comparable length. The highest‐scoring hit belonged to the crystal structure of *Burkholderia* AP3 phage endolysin AP3gp15 (PDB 5NM7_A; Maciejewska et al. 2017), and the second‐best hit was to an endolysin from *E. coli* O157:H7 phage FTEbC1, LysT84 (also found in *Salmonella* phage GEC_vB_GOT, PDB 7RUM_A; Love et al. 2022). Notably, while the latter (LysT84) was separated from the El1 described in this study by an extremely short patristic distance in a reconstructed phylogeny, and represents the same cluster as LysT84 and 27 other endolysins at 95% identity, the former (AP3gp15 endolysin) is a substantial patristic distance apart from either of them (Figure 6).

To further investigate the structural similarity, the El1 structure was predicted using the AlphaFold3 server (Abramson et al. 2024) with a predicted template modeling (pTM) score = 0.85 and predicted local distance difference test (plDDT) score of very high (> 90) for most of the structure (with some regions being “confident” (90 > plDDT > 70), while only the linker between the PG binding domain and the EAD actually dropped to “low” plDDT within the range of 70–50. This, we believe, could be expected, as the related structural homologs mentioned previously were actually likely part of the training data set for the recent models predicting protein structures based on the sequence data.

**Figure 7:**
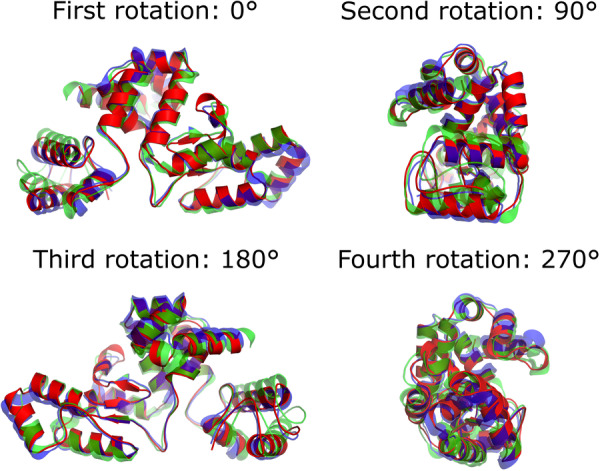
Structural comparison of an AlphaFold3‐predicted El1 endolysin model with experimentally determined crystallographic structures of homologous endolysins. The predicted structure of the *Escherichia* phage Sa157lw endolysin (El1) generated with AlphaFold3 (red) was structurally aligned with HHPred‐identified closely related endolysin structures from the Protein Data Bank, 7RUM (blue), and 5NM7 (green). Structures were superposed using Cα atoms in open‐source PyMOL v3.1.0. The models exhibited strong structural conservation, with backbone root‐mean‐square deviation values of ~0.996 Å (7RUM vs. AF3) and ~0.986 Å (5NM7 vs. AF3). Experimental structures are shown with partial transparency to highlight the overlap with the predicted model. Four different rotations around the horizontal axis (with a step of 90°) are shown for the three structures overlay.

When we loaded this structural prediction and both LysT84 and AP3gp15 to the open‐source PyMOL Molecular Graphics System (Version 3.1.0, Schrödinger, LLC) and removed the duplicate crystallographic chains for the experimentally determined structures, followed by an overlay using the “super” alignment algorithm in PyMOL to El1 in the case of each structure, the resulting root‐mean‐square deviation values were ~0.996 and ~0.986 Å, respectively (Figure 7). This, indeed, verified a very close structural conservation of the overall structure, expected in the case of El1 versus LysT84 comparison (both amino acid sequences differ in only at position 15 with Ala or Ser, and at position 28with Thr or Val, while otherwise having an identical native protein aa sequence), and, by extension, AP3gp15, based on the previously noted structural similarity of AP3gp15 and LysT84 (Love et al. 2022). This was further visually corroborated by representing the structural alignments in cartoon representations, highlighting secondary structure elements (Figure 7).

Thus, it can be concluded with confidence that El1, like LysT84 and AP3gp15, is a tightly packed α‐helical protein, in which the N‐terminal domain (PG‐binding domain) is separated from the C‐terminal domain (enzymatically active domain) by a flexible linker region. Although not further experimentally investigated within the scope of this work, such a degree of conservation suggests that even the features such as an active‐site residue positioning and the catalytic core of El1 could be much alike to those previously described earlier for LysT84 (Love et al. 2022) and AP3gp15 (Maciejewska et al. 2017). Given the overall sequence and structure similarity, we expect the catalytic region of El1 to function as proposed for the LysT84, with conserved Glu101 and Glu118 and Ser179 also being critical for catalysis of PG degradation (Love et al. 2022).

### 3.5 Effect of El1 on Other Gram‐Negative Bacteria

Finally, we assessed the bacteriolytic effect of El1 on a broader range of Gram‐negative cells using double‐layer agar plates as described in Section 2 (Table 1 and Figure S3). Although not all of the tested cells exhibited characteristic lysis spots, some of the targeted environmental strains were lysed by El1 in all the concentrations tested. Even an El1 concentration of 0.35 mg/mL was enough to ensure lysis for most of the susceptible strains. This effect was observable in a broad range of evolutionary divergent Gram‐negatives, which collectively represented several different orders of *Gammaproteobacteria*. Namely, the El1 was able to form a zone of clearance in the lawns of bacteria representing *Enterobacterales* (*Escherichia*, *Erwinia*, *Serratia*, and *Pantoea*), *Pseudomonadales* (*Pseudomonas* and *Acinetobacter*), *Aeromonadales* (*Aeromonas*), and *Lysobacterales* (*Stenotrophomonas*). While the qualitative nature of these spot assay observations that were registered as binary outcomes makes direct comparison of the effects on different bacteria hardly possible, representative examples of the lysis zones on the lawns of such bacteria as *E. coli*, *Aeromonas hydrophila*, *Aeromonas salmonicida*, *Acinetobacter calcoaceticus*, as well as *Erwinia billingiae* are given in Figure S3, and we believe they demonstrate an undeniable effect, quantification of which could be pursued in further studies. Collectively, the observations on the breadth of the susceptible target range of El1 let us carefully speculate that El1 could also be active against some medically important antibiotic‐resistant strains from the genera, environmental representatives of which were found susceptible. Thus, further studies of El1‐like endolysins could include assays against such relevant pathogens as UPEC, *P. aeruginosa*, *A. baumannii*, or *Stenotrophomonas maltophilia*. In case of at least a partial success against some of the strains representing these species, which are relevant in healthcare settings, artificial design for the improvement of the native El1‐like endolysins could prove itself a worthwhile endeavor.

**Table 1:** Reactivity of El1 against a broad range of Gram‐negative bacterial strains in a spot test. The results are qualitative and were registered in a binary form upon inspection of the corresponding strain bacterial lawn after an overnight incubation (+, lysis; −, no lysis; see Figure S3 for examples of the positive lawns).

	Endolysin El1, mg/mL			
Strain	2.8	1.4	0.7	0.35
*Escherichia coli* XL1‐Blue	+	+	+	+
*E. coli* JM109	+	+	+	+
*E. coli* TOP10	+	+	+	+
*E. coli* ClearColi BL21 (DE3)	+	+	+	+
*Aeromonas hydrophila* BIOR4288	+	+	+	+
*Aeromonas salmonicida* BIOR3022	+	+	+	+
*Acinetobacter calcoaceticus* AC1	+	+	+	+
*Erwinia billingiae* H6	+	+	+	+
*Pseudomonas folii* H40	+	−	−	−
*Pseudomonas orientalis* H27	+	+	+	−
*Pseudomonas kitaguniensis* H37	+	+	+	+
*Stenotrophomonas nematodicola* H46	+	+	−	−
*Pseudomonas lurida* H28	+	−	−	−
*Pseudomonas coleopterorum* H25	+	+	+	+
*Pantoea vagans* H62	+	+	+	+
*Pseudomonas neuropathica* H65	+	+	+	−
*Pseudomonas germanica* H67	+	+	+	+
*Pseudomonas canadensis* H43	+	+	+	+
*Pseudomonas sivasensis* H104	+	+	+	+
*Pseudomonas extremaustralis* H114	−	−	−	−
*Serratia bozhouensis* H57	−	−	−	−
*Pantoea agglomerans* H89	+	+	−	−
*Winslowiella iniecta* H24	+	+	+	+
*Pseudomonas meliae* H87	+	+	+	+
*Pseudomonas bubulae* H31	+	+	−	−
*Pseudomonas ficuserectae* H81	+	+	+	+
*Pseudomonas paralactis* H97	+	+	−	−
*Pseudomonas helleri* H33	+	+	+	−
*Tatumella ptyseos* H19	−	−	−	−
*Pantoea pleuroti* H111	−	−	−	−
*Pseudomonas avellanae* H79	+	+	+	−
*Acidovorax antarcticus* H45	+	+	+	+
*Silvania hatchlandensis* H30	−	−	−	−
*Pseudomonas trivialis* H39	−	−	−	−

Notably, El1 is not the first endolysin described to exhibit broad‐range lytic activity against different bacteria. For example, the antibacterial activity of *A. baumannii* phage ՓAB2 endolysin (LysAB2) has been reported (Lai et al. 2011) against both Gram‐positive and Gram‐negative bacteria. It was shown that treating bacteria with LysAB2 enhanced permeation of the cytoplasmic membrane of various bacteria, also to a different extent based on the targeted strain. The authors performed similar experiments to ours, including determination of the viable cell titers and registering changes in cell culture OD upon growth in the presence of an endolysin. However, the authors have used a panel of different reference cells and a much higher working concentration of LysAB2 (500 µg/mL) in comparison to El1 (20 µg/mL) used in this study. Therefore, the lytic properties of these two endolysins cannot be compared directly. Another very efficient example of a novel enzybiotic antibacterial is Artylisin Art‐175, shown to be active against *A. baumannii*, *P. aeruginosa*, and colistin‐resistant *E. coli* strains (Schirmeier et al. 2018). The authors of Art‐175 designed an artificial protein containing a sheep myeloid 29‐amino acid (SMAP‐29) peptide fused to the KZ144 endolysin. Again, direct comparison of the aforementioned endolysin activities is impossible due to the use of different strains in the efficiency assays. Without doubt, Art‐175 is a novel antibacterial that is well‐suited for a broad range of applications in hygiene, veterinary, and human medicine, importantly, one with the potential to target persister‐driven chronic infections. Despite the fact that previously developed Art‐175 has proven to be very efficient against a range of defined multidrug‐resistant pathogenic bacteria, the diversity of bacteria of potential economic importance urges us to look beyond the healthcare settings alone. Studying endolysin effects against a wider diversity of currently recognized culturable bacteria, while potentially perceived as being of mainly fundamental interest, suggests that the effects of each endolysin are extremely variable and there might still be a need for the development of new endolysin‐based antibacterials. El1, alone or possibly in combination with other endolysins, might represent another promising tool in the battle against unwanted Gram‐negative bacteria.

According to our current knowledge, this might be the first bactericidal characterization of an unmodified modular endolysin with El1‐like features that demonstrated extracellular activity against nonpermeabilized Gram‐negative bacterial cells. As the precise structure of the El1 is not yet elucidated experimentally, and no directed mutagenesis‐affected or truncated protein variant assays were performed, the exact mechanistic understanding of its mode of action against intact Gram‐negatives when applied exogenously still remains speculative. However, two experimentally determined structures from related proteins (LysT84 and AP3gp15), which show substantial structural conservation with the predicted structure of El1, represent an all‐α‐helix protein (Maciejewska et al. 2017; Love et al. 2022). Our current guess would be that one of these helices, either at an N‐, or C‐terminus, might act in a manner similar to antimicrobial peptides that can penetrate the outer membrane of Gram‐negatives and provide endolysin with access to its substrate, with the ensuing degradation of the PG layer leading to the death of an affected cell.

While incremental at this stage, we hope that our work on the El1 endolysin presented herein, as well as previous work on AP3gp15 and LysT84 by other researcher teams, will continue to inspire and facilitate further studies of El1‐like endolysins, which should eventually allow determining their possible worth in biocontrol applications against Gram‐negative pathogens of healthcare and economic importance and beyond.

## Author Contributions

**Tatjana Kazaka:** conceptualization, funding acquisition, project administration, supervision. **Nikita Zrelovs:** data curation, software, writing – original draft, writing – review and editing. **Inara Akopjana:** investigation, methodology. **Janis Bogans:** investigation, methodology. **Juris Jansons:** investigation, methodology. **Andris Dislers:** conceptualization, supervision, writing – review and editing. **Andris Kazaks:** conceptualization, data curation, project administration, supervision, writing – original draft, writing – review and editing.

## Ethics Statement

The authors have nothing to report.

## Conflicts of Interest

The authors declare no conflicts of interest.

## Supporting information

Supporting File 1

Supporting File 2

Supporting File 3

Supporting File 4

Supporting File 5

Supporting File 6

Supporting File 7
