# Supplementary figures and images for: Investigation of Recombinantly Produced Endolysins Reveals a Modular Enzyme Shared by Several Enterobacteria Phages to Exhibit Broad‐Range Lytic Activity Against Different Orders of Gammaproteobacteria
Source: Microbiologyopen. 2026 Apr 16;15(2):e70293. doi: 10.1002/mbo3.70293 (PMC13084259; doi:10.1002/mbo3.70293)

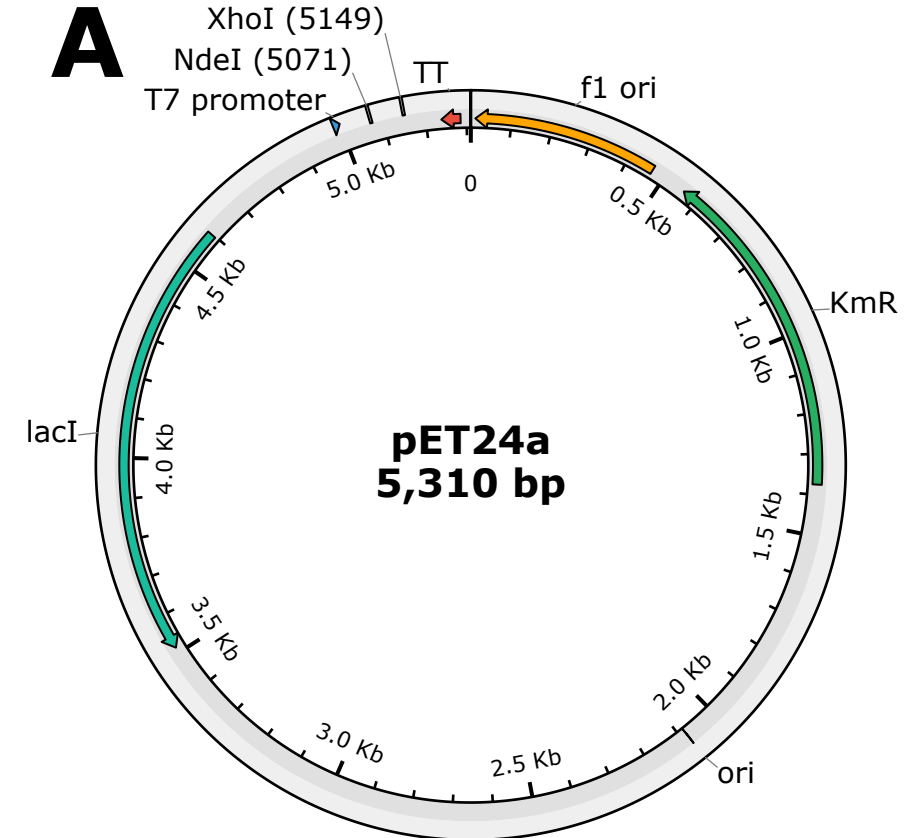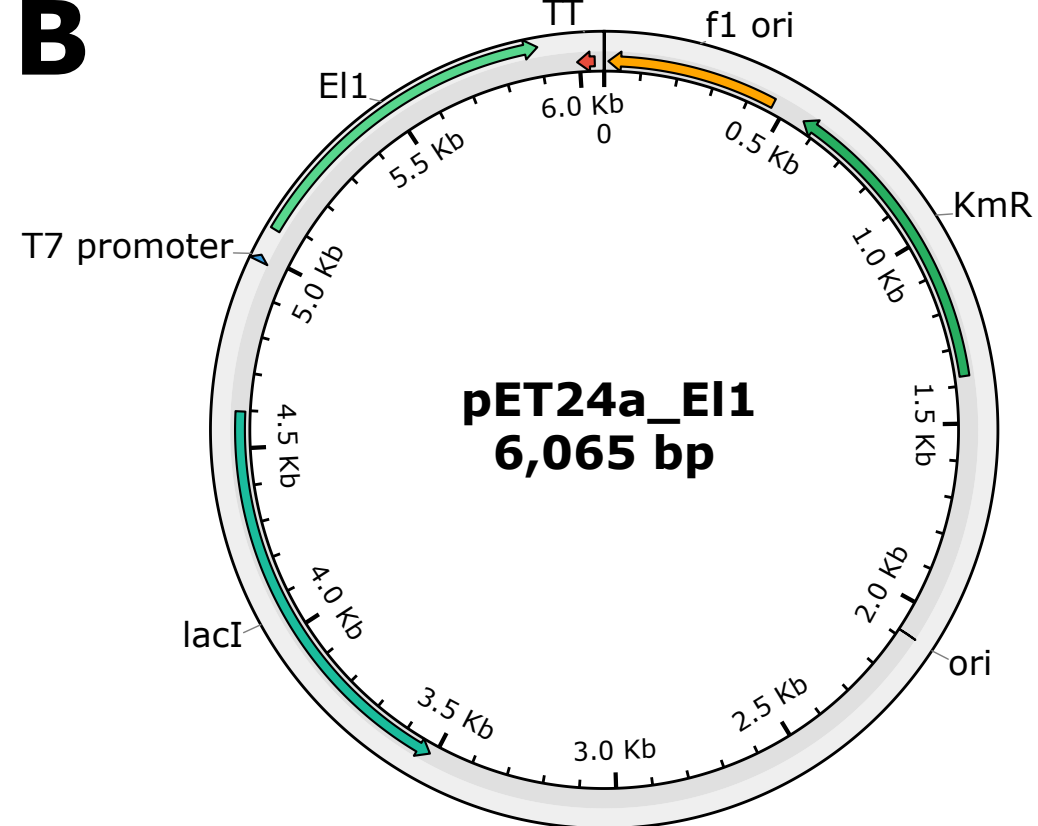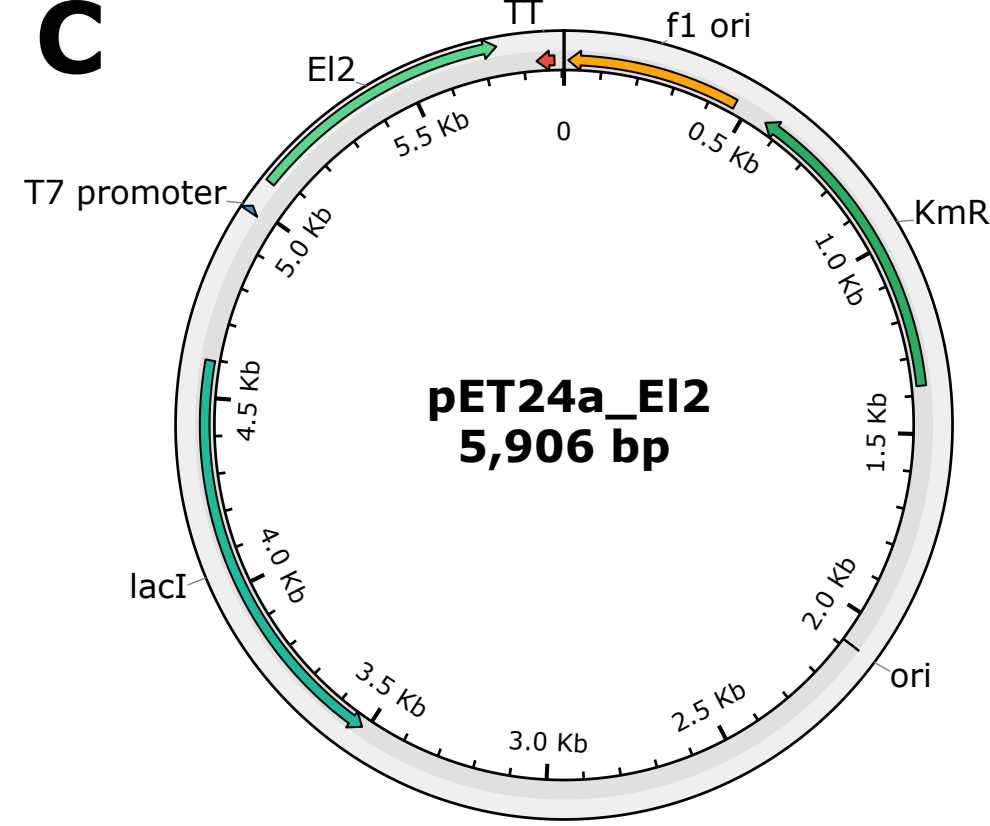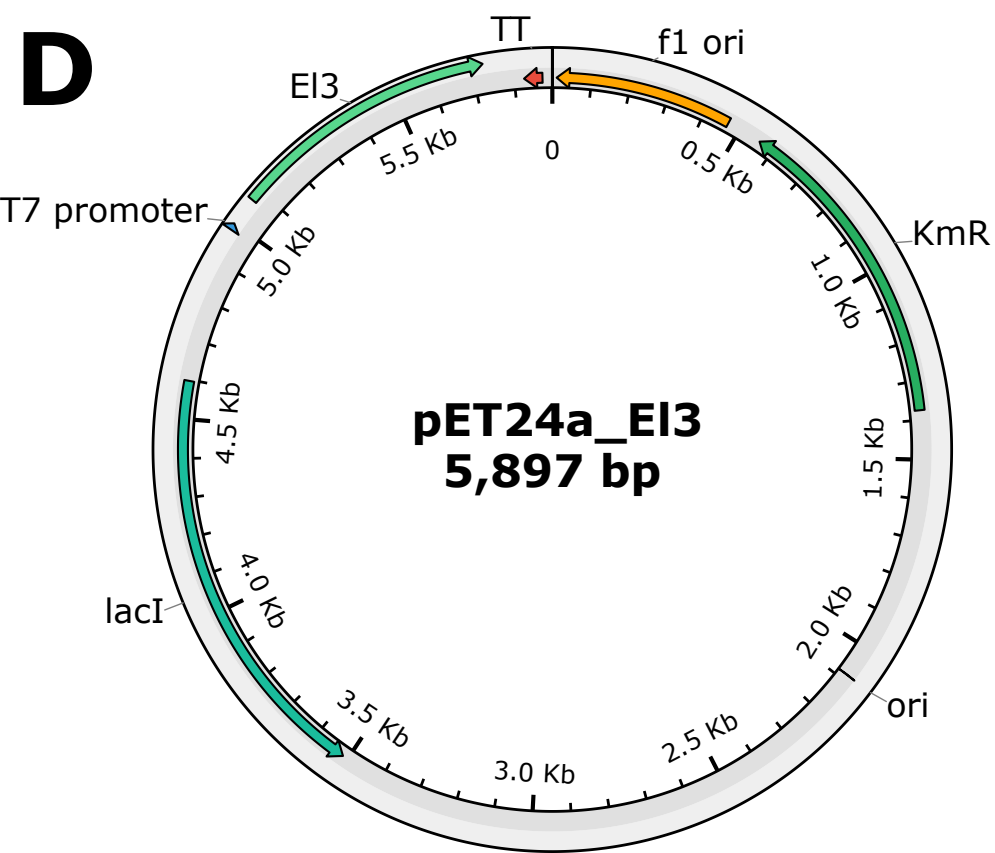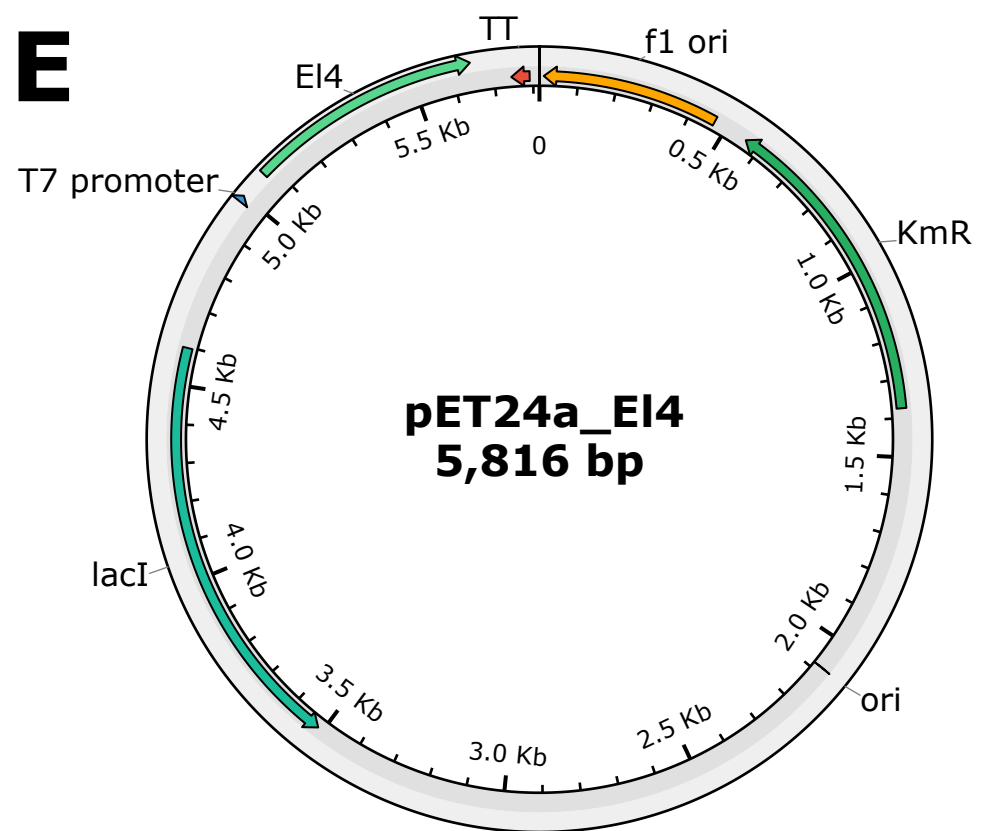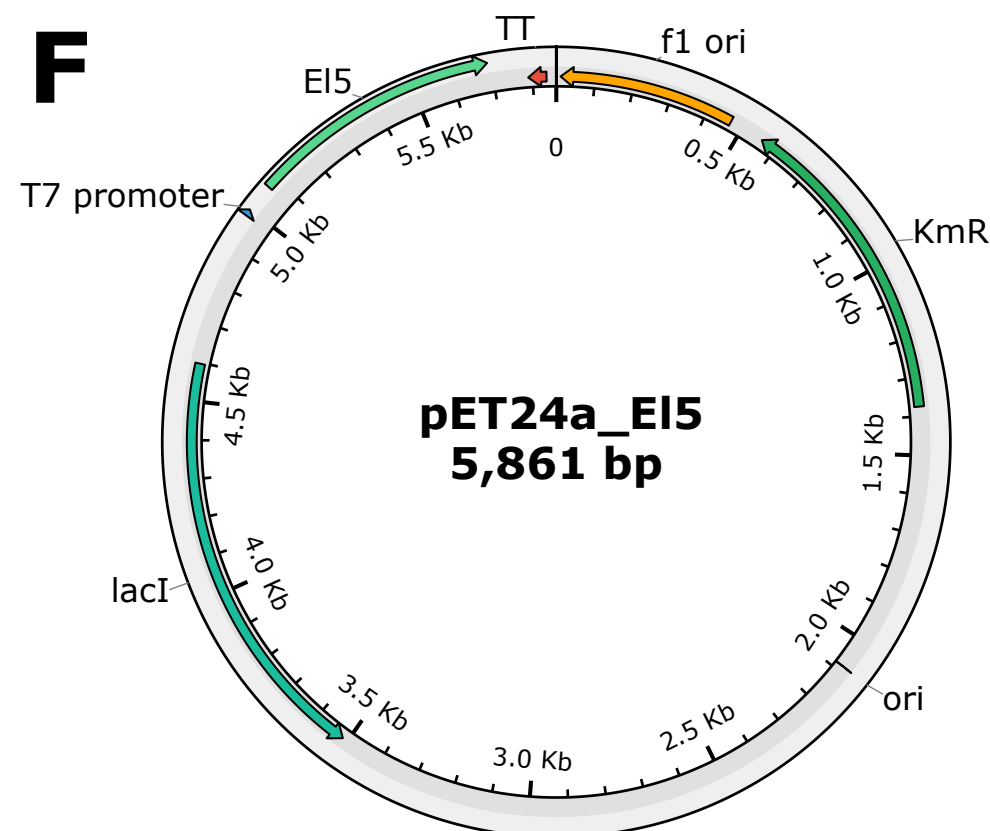

Supplement: Supplementary file 1 — Supporting File 1 [file MBO3-15-e70293-s006.pdf]

mg/mL

2.8

1.4

0.7

0.35

A

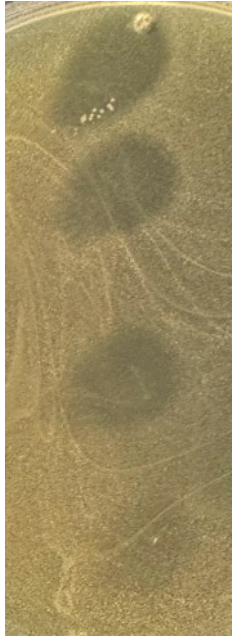

B

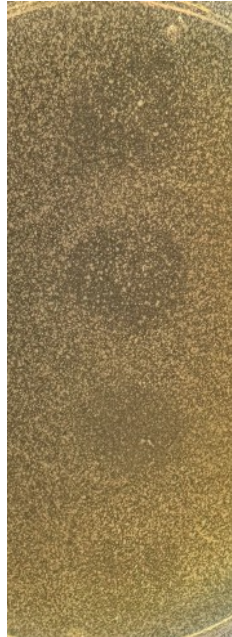

C

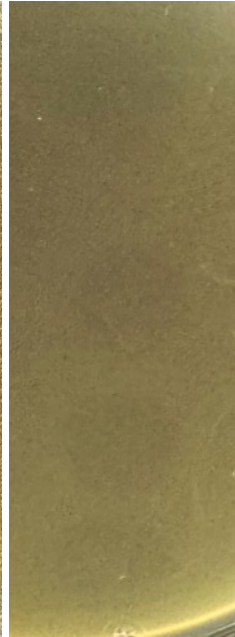

D

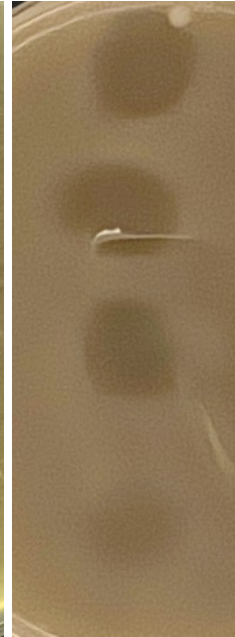

E

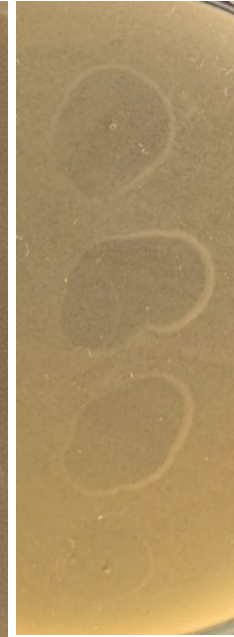

F

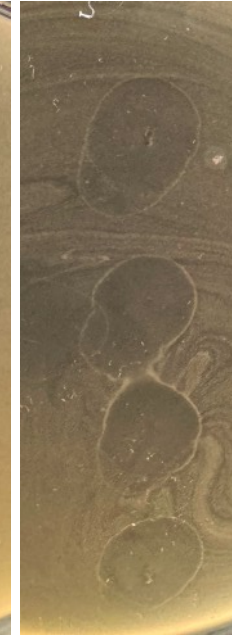

G

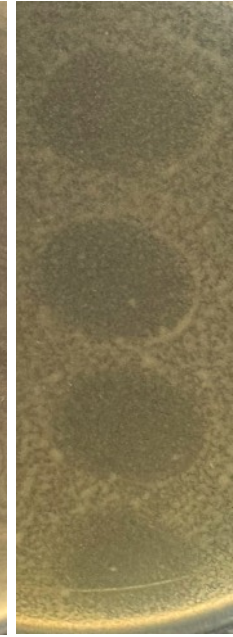

H

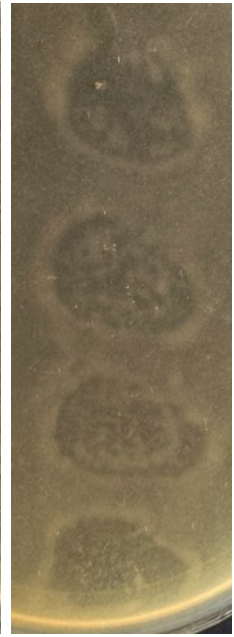

Supplement: Supplementary file 3 — Supporting File 3 [file MBO3-15-e70293-s005.pdf]
