# Supplementary material for: Investigation of Recombinantly Produced Endolysins Reveals a Modular Enzyme Shared by Several Enterobacteria Phages to Exhibit Broad‐Range Lytic Activity Against Different Orders of Gammaproteobacteria
Source: Microbiologyopen. 2026 Apr 16;15(2):e70293. doi: 10.1002/mbo3.70293 (PMC13084259; doi:10.1002/mbo3.70293)
Supplement: Supplementary file 2 — Supporting File 2 [file MBO3-15-e70293-s002.pdf]

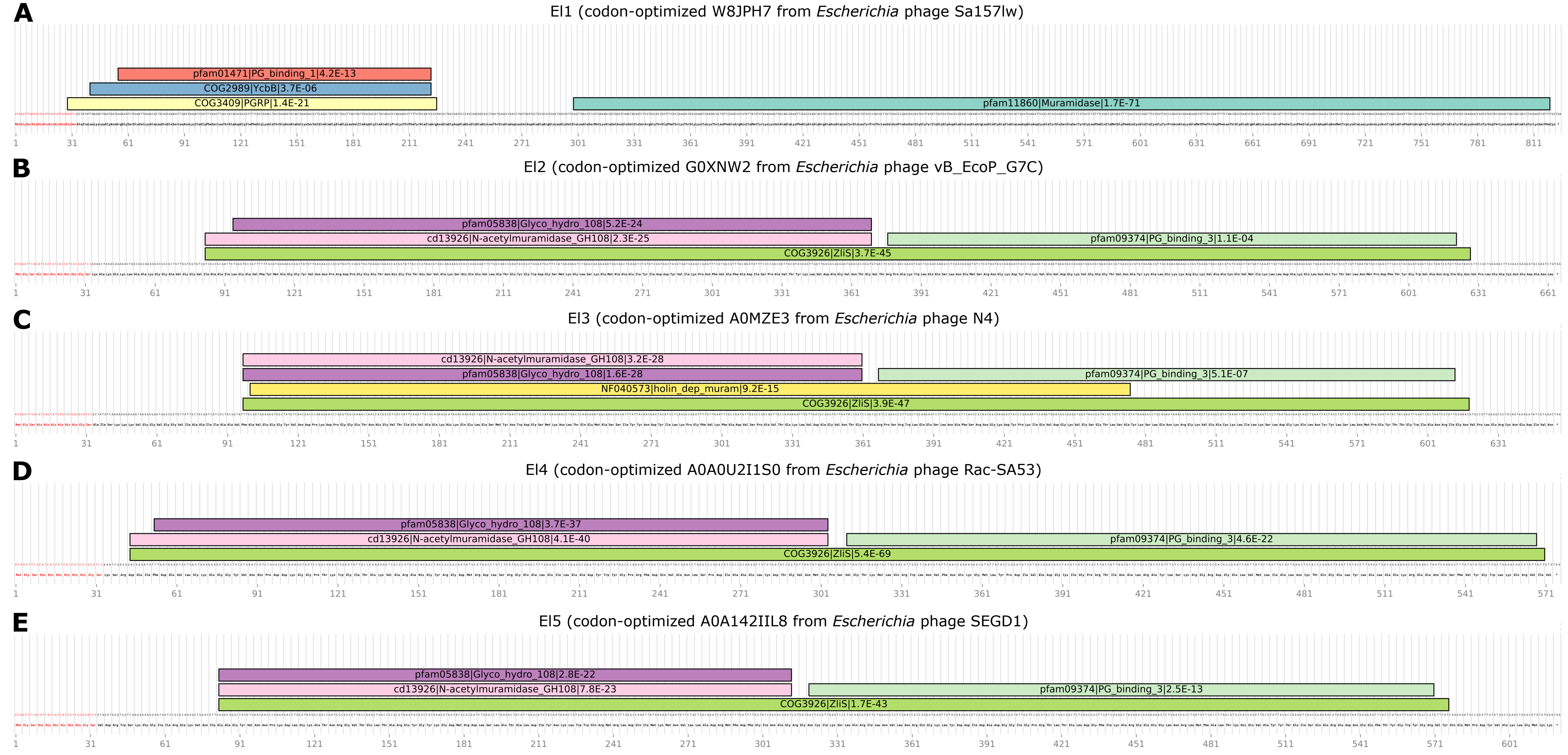
A
EI1 (codon-optimized W8JPH7 from *Escherichia* phage Sa157lw)
pfam01471|PG_binding_1|4.2E-13
COG2989|YcbB|3.7E-06
COG3409|PGRP|1.4E-21
pfam11860|Muramidase|1.7E-71
B
EI2 (codon-optimized G0XNW2 from *Escherichia* phage vB_EcoP_G7C)
pfam05838|Glyco_hydro_108|5.2E-24
cd13926|N-acetylmuramidase_GH108|2.3E-25
pfam09374|PG_binding_3|1.1E-04
COG3926|ZliS|3.7E-45
C
EI3 (codon-optimized A0MZE3 from *Escherichia* phage N4)
cd13926|N-acetylmuramidase_GH108|3.2E-28
pfam05838|Glyco_hydro_108|1.6E-28
pfam09374|PG_binding_3|5.1E-07
NF040573|holin_dep_muram|9.2E-15
COG3926|ZliS|3.9E-47
D
EI4 (codon-optimized A0A0U2I1S0 from *Escherichia* phage Rac-SA53)
pfam05838|Glyco_hydro_108|3.7E-37
cd13926|N-acetylmuramidase_GH108|4.1E-40
pfam09374|PG_binding_3|4.6E-22
COG3926|ZliS|5.4E-69
E
EI5 (codon-optimized A0A142IIL8 from *Escherichia* phage SEGD1)
pfam05838|Glyco_hydro_108|2.8E-22
cd13926|N-acetylmuramidase_GH108|7.8E-23
pfam09374|PG_binding_3|2.5E-13
COG3926|ZliS|1.7E-43
